# Does the Internet Moderate the Neighborhood Effect? Internet Use, Neighborhoods, and Mental Health among Older Adults in Shanghai

**DOI:** 10.3390/ijerph20032267

**Published:** 2023-01-27

**Authors:** Wei Chen, Jia Miao

**Affiliations:** 1 School of Sociology and Political Science, Shanghai University, Shanghai 200444, China; 2 Center for Applied Social and Economic Research (CASER), NYU Shanghai, Shanghai 200126, China

**Keywords:** neighborhood effects, internet use, digital divide, mental health, older adults

## Abstract

Internet use may reduce the impact of the neighborhood on residents’ well-being by helping people utilize resources beyond their immediate neighborhoods or strengthen neighborhood influences by widening the digital divide across neighborhoods. This study investigates how internet use moderates neighborhood effects on mental health among older adults in Shanghai. Using data from the Shanghai Urban Neighborhood Survey (SUNS) and population census, hierarchical linear models reveal that older adults who more frequently use the internet report lower levels of mental distress. Internet use attenuates the negative effects of living in low-socioeconomic status (SES) neighborhoods. We also examine the roles of three types of internet use: social networking, leisure, and information seeking. The results show that only social networking and leisure internet use are significantly associated with improved mental health among older adults. The results suggest that social programs are needed to increase internet literacy among older adults to promote active aging, and priority should be given to relatively disadvantaged neighborhoods.

## 1. Introduction

Population aging has become a formidable challenge facing many societies. The question of how to sustain the physical and mental health of a growing number of older adults has a significant bearing on sustainable social development. Extensive studies have revealed that neighborhood social and physical environments play pivotal roles in shaping the mental health of elderly people [1,2]. Due to retirement, loss of family members and friends, and decreased physical mobility, older adults are more profoundly affected by their immediate environments than are other adult populations [3,4]. Many governments have thus committed to building aging-friendly neighborhoods and helping elderly people “age in place”.

The importance of neighborhoods, however, may have changed in the digital era, as people can now conveniently extend their social networks and daily living activities far beyond their neighborhoods’ physical boundaries. Internet use can weaken or strengthen the role of the neighborhood in determining older adults’ well-being. On the one hand, the internet enables people to seek resources beyond their neighborhoods, as the rapid development of information and communication technology (ICT) has made the internet more accessible and affordable [5,6]. Therefore, neighborhood effects on mental health may gradually decrease in the foreseeable future. On the other hand, internet use may further highlight the role of the neighborhood in maintaining health. Across neighborhoods, there is a digital divide in ICT access, how it is used, and its effects [7,8,9]. Residents in higher socioeconomic status (SES) neighborhoods generally enjoy better internet access and use the internet more productively [7,9,10,11]. This is particularly true for elderly people, who encounter greater barriers to the internet and digital device use than young adults [12].

This study empirically tests these competing hypotheses by investigating how internet use moderates neighborhood effects on mental health among older adults in Shanghai, China. ICT has rapidly developed over the past decade in China, especially in megacities such as Shanghai. The number of internet users in China reached 1.05 billion by June 2022. There are approximately 119 million elderly internet users (people aged 60 years old and above) [13]. A recent survey revealed that about 81 percent of older adults aged 60 years old and above in Shanghai owned smartphones. However, only 32.4 percent of them reported that they could use smartphones without the assistance of other people [14]. Researchers have documented that internet use is closely related to health and subjective well-being among Chinese adults [8,15]. Nevertheless, they have rarely examined how internet use interacts with neighborhood SES and jointly shapes the health outcomes of older Chinese adults. This is largely because a neighborhood-based, population-representative dataset was not available until recently.

The current study uses data from the Shanghai Urban Neighborhood Survey (SUNS) and the Shanghai population census (aggregated at the neighborhood level). SUNS is a citywide representative survey providing comprehensive information about elderly people and their families. The census data enable us to capture neighborhood socioeconomic conditions. This study contributes to the literature by engaging in the heated debate on whether and how the internet has redefined the concept of “neighborhood.” By investigating how the association between neighborhood conditions and mental health varies according to internet use, this study can enhance our knowledge of the neighborhood effect in the information age. From the standpoint of public policy, it offers scientific evidence for measures aimed at promoting internet use among older adults in China. The internet has played an increasingly crucial role in supporting older adults, especially since the outbreak of COVID-19, a period in which the Chinese government has been determined to promote “Internet plus” elderly care services. The current study identifies some macro and micro level factors affecting older adults’ internet use, thus contributing to the formulation of social programs that will help the elderly embrace new technology and achieve active aging.

## 2. Literature Review

### 2.1. Neighborhood Effects on Mental Health

It has been well-documented that neighborhoods’ physical and social conditions are consequential to mental health [1,16,17,18]. In the context of the neighborhood, “physical” refers to the natural (e.g., air and noise) and built environments (e.g., green space and road networks), while “social” often refers to demographic and socioeconomic conditions, interpersonal interactions, social services, and social organizations [2]. Neighborhoods can directly influence mental health by placing stress on individuals. They can also exert indirect impacts by buffering the adverse consequences of stress by generating social support and networks [2].

Among the various neighborhood social conditions, neighborhood socioeconomic status (SES) has received extensive attention in empirical studies in the Western context. Neighborhood SES, typically measured by poverty, unemployment, and family wealth, is significantly positively associated with mental health [19,20]. Low-SES neighborhoods are generally characterized by a high resident turnover rate, low collective efficacy, and a greater number of neighborhood problems, which are related to an increased risk of depression among residents [21]. Moreover, the proportion of elderly people in a neighborhood has been found to influence the mental health of older adults. Elderly people are more likely to develop social connections and derive mutual support when there are more same-aged persons in their immediate environment [22].

### 2.2. Internet Use and Neighborhood-Mental Health Dynamics

The impact of internet use on social interaction may provide the most important mechanism for its moderation of neighborhood effects on mental health. Nevertheless, whether internet use can promote meaningful and beneficial social communication is still a subject of debate. Some scholars have argued that internet use fosters extended and higher-quality social networks by freeing people from the constraints of geography or isolation brought about by stigma or illness [5]. Many scholars have uncovered positive associations between internet use and social capital indicators, including social networking, civic participation, and volunteering [23,24,25,26,27]. They have found that despite the lack of face-to-face interaction, internet-based communication can have strong networking effects. Internet use allows people to participate in social groups on the basis of common interests rather than geographical proximity [28,29]. Within neighborhoods, internet use does not replace in-person communications. Instead, it enhances the interactions between residents by adding a new means of connecting across existing relationships [6].

Some scholars have contended that internet-based interaction allows only limited and superficial human communication. According to these scholars, internet use may cause people to become socially isolated and cut off from genuine social relationships as they access the internet over their terminals in solitude or communicate with anonymous strangers [15]. Greater internet use has been found to be associated with declines in interaction with family members, a reduction in the size of social networks, and increases in depression and loneliness [30]. The most obvious mechanism is time replacement. When people spend a substantial part of their time on-screen, this time is no longer available for other social activities and interactions [31].

A randomized, controlled intervention study showed that computer and internet use neither positively nor negatively influences individual well-being and mood or the social networks of healthy older adults [23]. Some scholars have claimed that arguments based on the attributes of the technology alone do not resolve this debate, as people may use the internet for many purposes, including communication, entertainment, and information seeking. When people use the internet primarily for entertainment and information, they may engage in limited face-to-face social interaction. By contrast, when people use the internet for interpersonal communication, their social networks may expand. However, social interactions and relationships on the internet may differ from traditional social communication. Thus, social uses of the internet may not have the same effects as traditional social activities [30].

Research in Western contexts has determined that older adults most commonly use the internet for communication and social support. Other common uses include entertainment and information seeking, particularly for health-related information [32]. However, in contrast to the abovementioned argument, researchers have found that older adults’ instrumental uses of the internet (e.g., information seeking), rather than their social uses, are related to their social well-being and that the association between different types of internet use and life outcomes varies according to individuals’ demographic or socioeconomic attributes [26].

### 2.3. Studies on China

In China, as the number of elderly internet users increases, a growing body of literature explores the impacts of internet use on the psychological well-being of the elderly. Previous studies have found that internet use is negatively associated with depression [33,34,35]. One important mechanism of this association is the improved social participation and increased social capital that accompanies internet use. Compared with non-users, elderly internet users report larger and more diverse network sizes and more active social participation [36,37,38]. Some scholars have claimed that real-world social interaction is not the mediator but rather the moderator of the association between internet use and mental health and that older adults who are more actively engaged in in-person interaction report higher levels of hopefulness and lower levels of loneliness and are less likely to use the internet excessively [39].

Different types of internet use may exert various impacts on elderly people’s well-being in China. A survey conducted among people aged 60 years old and above in Shanghai reported that older people mainly used the internet for e-payment services, chatting with family members and friends, and seeking and sharing information [14]. Using a national representative survey, scholars found that reading news, watching videos, and playing games online significantly improved the mental health of middle-aged and elderly people in rural China while chatting online and other internet activities did not benefit mental health status [36].

Existing studies have substantially enhanced our understanding of the association between internet use and mental health. However, few studies have investigated how internet use moderates the relationship between neighborhood and mental health in a Chinese context. A study in Shanghai revealed that worse neighborhood environments strengthened the correlation between internet use and mental distress [40]. However, this study measured neighborhood SES by the concentration of recreational places and institutions devoted to the elderly, a criterion that may not accurately reflect neighborhood social and economic conditions. Moreover, elderly people may use the internet in a self-selective manner. For instance, older adults who suffer from loneliness and mental distress may use the internet to supplement and increase their connections with others. Without adjusting for this potential selection bias, assessments of internet use on mental health are likely to be inaccurate.

## 3. Data, Measurement, and Methods

### 3.1. Data

This study uses data from the Shanghai Urban Neighborhood Survey (SUNS), which constitutes a unique, spatially constructed dataset for the study of the neighborhood effect in large Chinese cities [17]. The SUNS constructed a citywide representative sample of the population in Shanghai. It adopted multistage probability proportional to size sampling with implicit stratification. The main stratification variables included types of neighborhoods (downtown, new town, or suburban areas), socioeconomic development of sub-districts (jiedao), and proportion of internal migrants. All subsamples were obtained through three stages: the primary sampling unit was a sub-district or town (xiangzhen), the second-stage sampling unit was an urban neighborhood community (ju min wei yuan hui) or a village (cun min wei yuan hui), and the third-stage sampling unit was the household. The sampling units were selected using a systematic sampling method. The SUNS selected 5–13 sub-districts/towns from each district, from which 2 neighborhoods were selected. Thirty households were selected from each neighborhood, and all eligible members were interviewed face-to-face during home visits. All participants provided verbal informed consent. The baseline survey was conducted in 2016/17 and covered 180 neighborhoods, 5102 households, and 8631 adults aged 15 or above. The SUNS collected comprehensive information at the neighborhood, household, and individual levels..

We restrict the sample to adults aged 55 and above. In China, men and women are mandated to retire at 60 and 55, respectively. Because the term “elderly” is initially coined to describe retirees, we use age 55 as a cut-off point for older adults in our study. The SUNS successfully interviewed 3490 adults aged 55 years and older. After removing any observations with missing information for any individual- or neighborhood-level variables, our analytical sample consists of 2952 older adults in 174 neighborhoods.

We also use administrative data from the Population Office of the Shanghai Municipal Government, including the 2010 population census data and regularly updated registration records of both housing and the actual residential population. These datasets enable us to adequately measure neighborhood socioeconomic conditions. The administrative data are aggregated at the neighborhood level and matched with the SUNS data using unique official neighborhood codes.

### 3.2. Measurement

#### 3.2.1. Dependent Variable

We assess mental health with the Hopkins Symptom Checklist (HSCL-10), a tool widely used for both clinical and epidemiological purposes to detect psychological distress among adults [41]. HSCL-10 is a four-point Likert scale consisting of 10 items that measure two dimensions of mental distress: anxiety and depression. A higher score indicates worse mental health. The scale has high reliability, with a Cronbach’s alpha value of 0.89.

#### 3.2.2. Key Independent Variables

Internet use is measured by the frequency of internet access for one or more of four purposes: reading news, seeking health information, social networking, and leisure (i.e., playing games, watching TV, or listening to music). This measurement employs a four-item, five-point scale ranging from 1 (“never”) to 5 (“almost every day”). The average score of 4 items is used to measure overall respondent internet use. We also examine associations between various types of internet use and mental health. Reading news and seeking health information are combined into one category: “information seeking” (the average score of the two items is used).

Using data from the 2010 Shanghai population census (neighborhood-level aggregated data) and principal component analysis (PCA), we construct a neighborhood SES index to capture neighborhood SES conditions. Three indicators are used: the proportion of the population with a minimum of senior high school education, the proportion of the population in high-status occupations (i.e., managers, administrators, and professionals), and the proportion of the population holding an urban hukou (household registration). These three variables are combined to create a new variable. This variable’s eigenvalue reaches 2.6, and over 87% of its total variance can be explained by the three original socioeconomic variables with factor loadings of over 0.8. The principal component is then standardized so that each neighborhood is assigned a socioeconomic index ranging from 0 to 1 [17].

#### 3.2.3. Control Variables

We control for a set of individual attributes, including demographic characteristics, educational attainment, hukou type (local vs. non-local hukou), self-reported health status, employment status, and living with a household member younger than 55. At the neighborhood level, we control for the proportion of residents aged 60 and above and the location of the neighborhood in urban or non-urban districts.

### 3.3. Methods

The main purpose of this study is to investigate how internet use moderates the macro-level impact of neighborhood on mental health. To examine the cross-level interaction, we formulate a two-level hierarchical linear regression to estimate the association between neighborhood characteristics, internet use, and mental health. The regression is characterized by the following equations:

Level-1 Model (individual-level):

(1) $$\mathrm{Mental}\;\mathrm{Distress}=\pi_{0j}+\pi_{1j}Internet_{\begin{array}{c} ij\; \end{array}}+\Gamma X_{ij}$$

Level-2 Model (neighborhood-level):

(2) $$\pi_{0j}=\beta_{00}+\beta_{01}N\_SES_{j}+\delta W_{j}+r_{0j}$$

(3) $$\;\pi_{1j}=\beta_{10}+\beta_{11}N\_SES_{j}+r_{1j}$$

The level-1 model estimates respondents’ mental distress depending on a set of individual characteristics. $Internet_{ij}$ is the frequency of internet use reported by respondent *i* in neighborhood *j.* $X_{ij}$ is a vector of individual-level control variables.

The level-2 model assesses the heterogeneity in experiences of mental distress across neighborhoods and determines how internet use interacts with neighborhood SES and thus affects mental health. The impact of the internet, $\pi_{1j}$, is divided into two components: the mean effect across all neighborhoods ($\beta_{10}$) and the neighborhood-specific effect of internet use. The latter also consists of three components: the effect related to neighborhood SES ($\beta_{11}$), and the random effect associated with each neighborhood ($r_{1j}$). In the same way, the mean level of individual mental health $\pi_{0j}$ is modeled as neighborhood mean mental health ($\beta_{00}$) plus mental health variation determined by neighborhood SES ($\beta_{01}$) and other neighborhood characteristics (*δ*$W_{j}$), and variation ($\gamma_{0j}$) not captured by the multilevel model.

Internet use may be a selective practice. For instance, older adults who face shrinking social networks and deteriorating mental health may turn to the internet to supplement their interpersonal communication. To adjust for potential selection bias, we use the inverse probability of treatment weighting (IPTW). IPTW employs the propensity score to balance individual characteristics across internet users and non-users by the inverse probability of internet use. IPTW involves two main steps. First, the propensity toward internet use is calculated based on the individual’s characteristics (i.e., propensity score). Second, weights for each respondent are calculated as the inverse of the propensity score. The application of these weights to a study population creates a pseudopopulation in which confounders are equally distributed across internet users and non-users [42]. In this study, we estimate the propensity score using a logistic regression model to regress the indicator variable denoting internet use on a set of individual- and neighborhood-level variables listed in Table 1. Weights are calculated for each respondent as 1/propensity score for the internet users and 1/(1 − propensity score) for the non-users. After calculating the weights, we incorporate them into two-level hierarchical linear regression models to obtain estimates that are adjusted for selection bias.

## 4. Empirical Results

### 4.1. Descriptive Statistics

The average age of the respondents is 65.9 years old. More than 81 percent are local residents (i.e., hold a local *hukou*). The prevalence of depression among older adults in Shanghai is around 13%, when 1.85 is used as the cut-off value for predictions of depression [41]. This result is comparable with that of the Chinese Longitudinal Aging Study (CLAS), which found that 12.1% of adults in Shanghai aged 60 years and above suffered from depression [43]. Our sample shows that about 46 percent of older adults in Shanghai use the internet. This proportion is lower than that in the U.S. (67% [44]) but much higher than the national average in China (6.7% [8]).

The two-way tests in Table 1 show that internet users report better mental health than non-users, and the difference is statistically significant (two-tailed *t*-test, *p* < 0.001). Compared with non-users, elderly internet users are significantly younger, better educated, healthier, and more likely to live with household members younger than 55. There are also significant differences in neighborhood characteristics between internet users and non-users. Elderly users are more likely to live in higher SES neighborhoods with younger population age structures, indicating unequal access to the internet across neighborhoods in Shanghai.

### 4.2. Who Is Using the Internet? Evidence from Hierarchical Linear Models

To further investigate individual and neighborhood characteristics that are relevant to older adults’ internet use, we conduct a two-level hierarchical linear regression analysis. Model 1 in Table 2 reveals that younger, healthier, and better-educated older adults use the internet more frequently. People who are still active in the labor market and who live with young household members report a lower frequency of internet use than their counterparts. Employment and domestic work may have occupied substantial amounts of their time, which reduces the time that could have been spent on screens.

Model 2 shows that, when controlling for individual attributes, neighborhood SES is positively associated with the frequency of internet use. The results indicate that neighborhood conditions independently contribute to internet use among the elderly in Shanghai. Our model explains 75.57% of the variance in internet use across neighborhoods and 48.23% of the variance between individuals. The results suggest that neighborhood SES is the main determinant of the digital divide across neighborhoods. Model 3 treats internet use as a dichotomous variable, with 1 representing “[respondent has] ever used the internet”. It reveals generally consistent results. We find that the proportion of the elderly in the neighborhood is negatively associated with internet use by older adults. This result suggests that when older adults begin to use the internet, they may require supportive environments or assistance from younger persons to overcome psychological and technological barriers.

### 4.3. Internet Use, Neighborhood SES, and Mental Health

Table 3 shows the association between neighborhood SES, internet use, and mental distress among the elderly. Model 1 reveals that the frequency of internet use is negatively associated with mental distress among older adults. For instance, when an older adult changes from a non-user to an occasional user, his/her mental distress reduces by 0.16 standard deviations. This improvement is significant and substantial. We also find that, when controlling for older adults’ individual characteristics, neighborhood SES is positively associated with mental health. Higher-SES neighborhoods suffer from fewer social problems and can provide better facilities and social services, as well as more accessible and safer environments, thus significantly benefiting residents’ well-being.

**Figure 1:**
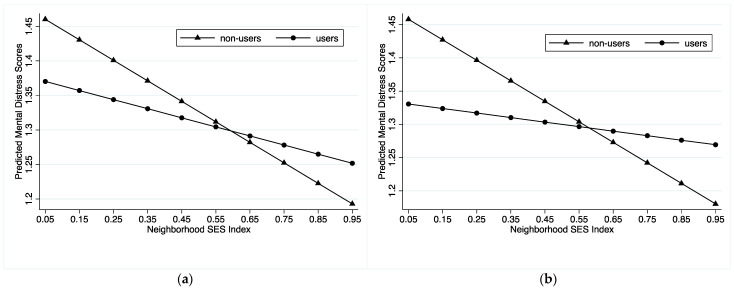
Neighborhood SES and Mental Distress: Internet Use as a Moderator.

The association between neighborhood SES and mental health is weaker for internet users than for non-users (Model 2). This indicates that internet use can moderate neighborhood SES-mental health dynamics by reducing the determining role of neighborhood SES. Model 3 presents the results of hierarchical linear models with IPTW. The moderating role of internet use becomes more prominent when we adjust for selection bias. The results suggest that certain unobserved variables, such as cognitive skills, simultaneously and positively affected internet use and mental health among the elderly. When we rule out the influence of the confounders, the effects of internet use increase. Figure 1a,b visualize the differences between Model 2 and Model 3. They show that although older adults in more affluent neighborhoods enjoy better mental health, internet use moderates this association. The influence of internet use is typically underestimated without correction of the selective bias.

### 4.4. Internet Use, Neighborhood SES, and Depression: By Purpose of Use

People use the internet for various purposes, which may lead to different mental health outcomes. Table 4 shows that when older adults use the internet for social networking and leisure, they report better mental health than non-users. Social use of the internet can help older adults to contact families and friends beyond the neighborhood, which reduces feelings of loneliness and isolation. This is particularly important for older adults suffering from physical function decline. Leisure activities can benefit overall well-being, as leisure encourages positive feelings, including a sense of purpose and meaning. Leisure activities also promote a variety of social and physical practices that allow individuals to feel refreshed and to better cope with stress.

We also find that the use of the internet for information seeking is unrelated to the mental status of older adults. One possible reason is that such activity could occupy time that may otherwise have been spent on social interactions or leisure. Moreover, scholars have found that older adults often use the internet to search for health-related information [32] indicating that when they use the internet for information purposes, they are likely facing certain health issues. Concerns about health conditions may place additional psychological burdens on the elderly.

## 5. Discussion

Many older adults wish to remain in their homes and neighborhoods and thus to “age in place”. Studies have revealed that aging in place can reduce public expenditures on health services and long-term care [45]. Internet use and ICT have opened opportunities to make older adults’ homes and neighborhoods safer, more comfortable, and more accessible [46]. However, older adults face unique barriers to fully realizing these opportunities. Compared with younger adults, the elderly are less familiar with information technology. This unfamiliarity may cause anxiety, fear, and feelings of low confidence, especially for older adults living in low-SES neighborhoods. Previous studies on Western populations have found that low-SES neighborhoods suffer from limited access to public services and facilities, as well as low levels of social trust, social interaction, and collective efficacy [47,48]. Internet use can help older adults to reach out to social contacts beyond the physical boundaries of the neighborhood, thus potentially improving their mental health [34,46].

Promoting equal internet access and internet literacy among the elderly has become more urgent since the outbreak of the COVID-19 pandemic. The internet has served as a vital platform for seeking information, deriving social support, receiving services, and purchasing basic commodities during the public health crisis [49]. The elderly population is the most vulnerable age group affected by the pandemic. Moreover, they are in a disadvantageous position when accessing and effectively using the internet. To safeguard this vulnerable population from COVID-19-like disasters in the future, we need to prepare them adequately for the rapidly evolving, digitally mediated world.

Recent studies reveal that the internet has been reshaping healthcare delivery during the COVID-19 pandemic by amplifying the role of telehealth and telemedicine. As the internet has played an increasingly important role in health access, digital access has been considered a social determinant of health [50]. This shift has profound implications for mental health inequality. The demand for mental health services has surged in the wake of the pandemic. A systematic review study noted that the general public reported lower psychological well-being and higher scores of anxiety and depression compared to before COVID-19. COVID-19 patients reported a high level of post-traumatic stress symptoms and depression [51]. Therefore, one can expect that internet would play an increasingly vital role in shaping mental health disparity among young and older adults.

This study finds that neighborhood conditions independently contribute to internet use and mental health among older people. In order to improve psychological well-being and reduce mental health inequality in Chinese cities, urgent actions are needed to address the current gaps in internet access and internet literacy. First, a mapping project is needed to identify broadband coverage gaps across neighborhoods and help disadvantaged neighborhoods to improve broadband internet infrastructure. Second, the government can provide subsidies for broadband subscribership, mobile health applications, and digital devices to help low-income people in low-SES neighborhoods to tackle mental health problems. Third, healthcare providers can partner with neighborhood-based organizations to deliver skills-building programs to improve digital and health literacy among older adults.

## 6. Conclusions

The internet plays an increasingly essential role in shaping our lives and well-being. However, there is a digital divide between people from diverse backgrounds and different neighborhoods. In this study, we find that neighborhoods exert independent impacts on internet use among the elderly in Shanghai. Older adults who enjoy higher social and health status and live in more affluent neighborhoods are more likely to benefit from the rapid development of technology. Older adults who use the internet more frequently report lower levels of mental distress than infrequent- and non-users. Moreover, internet use mitigates the negative effects of living in low-SES neighborhoods. Older adults in disadvantaged neighborhoods benefit from internet use more than their counterparts in high-SES neighborhoods. We also examine the roles of three types of internet use: social networking, leisure, and information seeking. The results show that only social networking and leisure internet use are significantly associated with reduced mental distress among older adults.

This study has certain limitations. First, COVID-19 pandemic has profoundly changed the way people use the internet and daily life in neighborhoods, which may alter the association between internet use, neighborhood SES, and mental health. Future studies may explore these changes using data collected after the outbreak of COVID-19. Second, due to the absence of relevant measurements in the data, we are unable to empirically test channels through which internet use affects mental health among older people. Future research may consider a more comprehensive dataset to investigate social mechanisms. Third, this study focuses on mental health. Internet use and neighborhood can affect behavioral health, which has wider policy implications. Further research is needed to address this issue.

Notwithstanding these limitations, this study provides rigorous empirical evidence to evaluate the role of the internet in promoting mental health and reducing neighborhood inequality. Our findings suggest that to promote active aging and aging in place, social programs are needed to increase internet literacy among older adults, and priority should be given to relatively disadvantaged neighborhoods.

## Figures and Tables

**Table 1:** Descriptive statistics of older adults in Shanghai: SUNS 2016/17.

Variables	Whole Sample	By Internet Use		
		Non-Users	Users	Diff
Individual-level				
Mental distress (1–4 points)	1.346	1.417	1.279	0.148 ***
	(0.492)	(0.545)	(0.415)	
Internet use (1–5 points)	2.092			
	(1.387)			
Internet use by purpose				
Social networking	2.132			
	(1.641)			
Leisure	1.918			
	(1.399)			
Information seeking	2.142			
	(1.457)			
Age	65.946	67.874	63.736	4.137 ***
	(7.712)	(8.276)	(6.328)	
Male	0.487	0.469	0.507	−0.037 *
	(0.500)	(0.499)	(0.500)	
Married (Yes = 1)	0.854	0.827	0.884	−0.057 ***
	(0.353)	(0.378)	(0.320)	
Local *Hukou* (Yes = 1)	0.814	0.814	0.815	0.001
	(0.388)	(0.389)	(0.389)	
Completed senior high school (Yes = 1)	0.411	0.209	0.642	−0.433 ***
	(0.492)	(0.407)	(0.480)	
Self-reported health status (1–5 points)	2.176	2.099	2.264	−0.165 ***
	(0.878)	(0.921)	(0.817)	
Active in the labor market (Yes = 1)	0.208	0.210	0.207	0.003
	(0.406)	(0.407)	(0.405)	
Younger adults in household (Yes = 1)	0.419	0.385	0.457	−0.071 ***
	(0.493)	(0.487)	(0.498)	
Neighborhood-level				
Neighborhood SES (0–1 point)	0.409	0.325	0.506	−0.181 ***
	(0.211)	(0.213)	(0.161)	
Urban district (Yes = 1)	0.663	0.488	0.864	−0.376 ***
	(0.473)	(0.500)	(0.343)	
% population aged 60+	0.207	0.212	0.203	0.009 ***
	(0.071)	(0.082)	(0.056)	
*N*	2952	1513	1439	

Note: Standard deviations are in parentheses. The significance of the difference between internet users and non-users is assessed by the two-tailed *t*-test. *** *p* < 0.001, * *p* < 0.05. Number of neighborhoods is 174.

**Table 2:** Coefficients of multilevel linear regression and logit models of individual and neighborhood characteristics on internet use among older adults in Shanghai: SUNS 2016/17.

Effects and Variables	Model 1	Model 2	Model 3
	HLM	HLM	Logit
**Fixed Effects**			
Individual-level predictors			
Age	−0.039 ***	−0.040 ***	−0.107 ***
	(0.003)	(0.003)	(0.008)
Male	−0.006	0.006	0.192 *
	(0.041)	(0.041)	(0.096)
Married (Yes = 1)	−0.002	−0.010	0.035
	(0.059)	(0.059)	(0.136)
Local *Hukou* (Yes = 1)	−0.025	−0.031	−0.032
	(0.081)	(0.080)	(0.172)
Completed senior high school (Yes = 1)	0.869 ***	0.778 ***	1.485 ***
	(0.045)	(0.046)	(0.098)
Self-reported health status	0.063 **	0.050 *	0.104 ^+^
	(0.024)	(0.023)	(0.054)
Active in the labor market (Yes = 1)	−0.128 *	−0.067	−0.126
	(0.056)	(0.056)	(0.135)
Younger adults in household (Yes = 1)	−0.182 ***	−0.196 ***	−0.276 **
	(0.043)	(0.043)	(0.098)
Neighborhood-level predictors			
Neighborhood SES		1.832 ***	3.517 ***
		(0.258)	(0.424)
Urban district (Yes = 1)		0.042	0.484**
		(0.114)	(0.178)
% population aged 60+		−0.263	−2.674 ***
		(0.416)	(0.716)
**Random Effects**			
Intercept	4.319 ***	3.696 ***	4.941 ***
	(0.241)	(0.248)	(0.567)
% Between-neighborhood variance explained		75.57	
% Between-individual variance explained		48.23	
R2 for overall model	0.233	0.312	0.292
AIC	9026.451	8914.4	
Number of individuals	2952	2952	2952
Number of communities	174	174	

Note: Standard errors are in parentheses. *** *p* < 0.001, ** *p* < 0.01, * *p* < 0.05, ^+^ *p* < 0.1.

**Table 3:** Coefficients of multilevel linear regression of neighborhood characteristics and internet use on mental distress among older adults in Shanghai: SUNS 2016/17.

Effects & Variables	Model 1 HLM	Model 2 HLM	Model 3 HLM-IPTW
**Fixed Effects**			
Neighborhood-level predictors			
Neighborhood SES	−0.351 ***	−0.297 **	−0.309 *
	(0.100)	(0.101)	(0.137)
Urban district (Yes = 1)	−0.002	−0.030	−0.027
	(0.045)	(0.040)	(0.051)
% population aged 60+	0.130	0.008	−0.201
	(0.160)	(0.143)	(0.179)
Individual-level predictors			
Internet use	−0.079 ***	−0.098 *	−0.140 *
	(0.020)	(0.046)	(0.061)
Age		−0.000	0.000
		(0.001)	(0.001)
Male		−0.141 ***	−0.116 ***
		(0.017)	(0.023)
Married (Yes = 1)		−0.118 ***	−0.145 ***
		(0.024)	(0.033)
Local *Hukou* (Yes = 1)		−0.059 ^+^	−0.037
		(0.032)	(0.035)
Completed senior high school (Yes = 1)		−0.061 **	−0.067 **
		(0.020)	(0.021)
Self-reported health status		−0.182 ***	−0.159 ***
		(0.010)	(0.012)
Active in the labor market (Yes = 1)		0.011	−0.009
		(0.023)	(0.029)
Younger adults in household (Yes = 1)		0.007	−0.010
		(0.017)	(0.018)
Cross-level interaction			
Neighborhood SES * Internet use		0.165 ^+^	0.241 *
		(0.094)	(0.120)
**Random Effects**			
Intercept	1.502 ***	2.136 ***	2.104 ***
	(0.040)	(0.103)	(0.125)
% between-neighborhood variance explained			22.73
% between-individual variance explained			28.00
R2 for overall model	0.041	0.186	0.270
AIC	4046.036	3592.123	6183.649
Number of individuals	2952	2952	2952
Number of communities	174	174	174

Note: Standard errors are in parentheses. *** *p* < 0.001, ** *p* < 0.01, * *p* < 0.05, ^+^ *p* < 0.1.

**Table 4:** Coefficients of multilevel linear regression of internet use on mental distress among older adults in Shanghai: SUNS 2016/17: By different purposes of use.

Effects and Variables	Social Networking			Leisure			Information Seeking		
	Model 1a	Model 1b	Model 1c	Model 2a	Model 2b	Model 2c	Model 3a	Model 3b	Model 3c
	HLM	HLM	HLM-IPTW	HLM	HLM	HLM-IPTW	HLM	HLM	HLM-IPTW
**Fixed Effects**									
Individual-level predictors									
Internet use	−0.043 *	−0.093 ^+^	−0.144 **	−0.054 **	−0.126 *	−0.188 ***	−0.026	−0.095 *	−0.132 *
	(0.020)	(0.056)	(0.051)	(0.020)	(0.051)	(0.050)	(0.020)	(0.048)	(0.065)
Neighborhood-level predictors									
Neighborhood SES	−0.208 *	−0.242 *	−0.225 ^+^	−0.203 *	−0.255 **	−0.314 **	−0.220 *	−0.284 **	−0.253 ^+^
	(0.092)	(0.098)	(0.129)	(0.091)	(0.097)	(0.112)	(0.091)	(0.100)	(0.134)
Cross-level interaction									
Neighborhood SES * Internet use		0.103	0.185^+^		0.154	0.270 **		0.151	0.201
		(0.107)	(0.105)		(0.100)	(0.098)		(0.096)	(0.124)
**Random Effects**									
Intercept	2.126 ***	2.137 ***	2.066 ***	2.143 ***	2.160 ***	2.127 ***	2.109 ***	2.129 ***	2.122 ***
	(0.101)	(0.102)	(0.129)	(0.101)	(0.102)	(0.123)	(0.101)	(0.102)	(0.123)
R2 for overall model	0.186	0.186	0.272	0.187	0.188	0.293	0.185	0.186	0.256
AIC	3590.233	3591.315	6060.763	3587.403	3587.071	5984.999	3593.12	3592.65	6315.655
Number of individuals	2952	2952	2952	2952	2952	2952	2952	2952	2952
Number of communities	174	174	174	174	174	174	174	174	174

Note: Standard errors are in parentheses. Controls are age, male, married, local *hukou*, completed senior high school, active in the labor market, younger adult in household, self-reported health status, urban district, and percentage of people aged 60 and above in the neighborhood. *** *p* < 0.001, ** *p* < 0.01, * *p* < 0.05, and ^+^ *p* < 0.1.

## Data Availability

Restrictions apply to the availability of these data. Data was obtained from Shanghai University and are available for use with the permission of Shanghai University.
